# Misdiagnosed HIV infection in pregnant women initiating universal ART in South Africa

**DOI:** 10.7448/IAS.20.7.21758

**Published:** 2017-08-29

**Authors:** Nei-yuan Hsiao, Allison Zerbe, Tamsin K. Phillips, Landon Myer, Elaine J. Abrams

**Affiliations:** ^a^ Division of Virology, Department of Pathology, University of Cape Town and National Health Laboratory Service, Cape Town, South Africa; ^b^ ICAP, Columbia University, Mailman School of Public Health, New York, NY, USA; ^c^ Division of Epidemiology and Biostatistics and Centre for Infectious Disease Epidemiology and Research, School of Public Health and Family Medicine, University of Cape Town, Cape Town, South Africa; ^d^ College of Physicians & Surgeons, Columbia University, New York, NY, USA

**Keywords:** HIV, diagnosis, rapid diagnostic test, specificity, ART, false positive, ELISA

## Abstract

**Introduction**: Rapid diagnostic tests (RDTs) are the primary diagnostic tools for HIV used in resource-constrained settings. Without a proper confirmation algorithm, there is concern that false-positive (FP) RDTs could result in misdiagnosis of HIV infection and inappropriate antiretroviral treatment (ART) initiation, but programmatic data on FP are few.

**Methods**: We examined the accuracy of RDT diagnosis among HIV-infected pregnant women attending public sector antenatal services in Cape Town, South Africa. We describe the proportion of women found to have started on ART erroneously due to FP RDT results based on pre-ART viral load (VL) testing and enzyme-linked immunosorbent assay (ELISA).

**Results**: We analysed 952 consecutively enrolled pregnant women diagnosed as HIV infected based on two RDTs per local guideline and found 4.5% (43/952) of pre-ART VL results to be <50 copies/ml. After excluding 6 women who had detectable virus on subsequent VL measurements, ELISA was performed on the 37 remaining women. Of these, 3/952 (0.3%) HIV RDT diagnoses were found to be FP. We estimate that using ELISA to confirm all positive RDTs would cost $1110 (uncertainty interval $381–$5382) to identify one patient erroneously initiated on ART, while it costs $3912 for a lifetime of antiretrovirals with VL monitoring for one person.

**Conclusions**: Compared to the cost of confirming the RDT-based diagnoses, the cost of HIV misdiagnosis is high. While testing programmes based on RDT should strive for constant quality improvement, where resources permit, laboratory confirmation algorithms can play an important role in strengthening the quality of HIV diagnosis in the era of universal ART.

## Introduction

Rapid diagnostic tests (RDTs) detecting the HIV-1/2 antibodies are used globally to diagnose HIV infection. When performed optimally, RDTs are highly sensitive and specific. In a World Health Organization (WHO) report of HIV assays, laboratory studies evaluating eight RDTs observed a sensitivity of 99.4–100% and a specificity of 98.9–100% [1]. In addition to their comparable performance with the gold standard enzyme-linked immunosorbent assay (ELISA), RDTs are inexpensive, are easy to use and can be used at point-of-care. With recent evidence and recommendations favouring early antiretroviral treatment (ART) initiation [2–4] and the universal “test-and-start” approach [5], the use of RDTs to quickly diagnose HIV infection and facilitate immediate ART initiation will be critical in achieving the UNAIDS 90-90-90 goal in many low- and middle-income countries (LMIC).

However, the quality of RDT diagnostic services is highly dependent on user training and quality assurance of the performing facility. The HIV testing services (HTS) are often overburdened with the high service load and lack the necessary training in quality assurance. As a result, the high sensitivity and specificity of RDT observed in assay evaluation studies may not translate to the same performance in real-world HTS. For example, according to a 2012 report, the level of testing process compliance among a sample of 38 South African health facilities was 3.4% with completion of registers, appropriate incubation time and post-test counselling cited as steps with the poorest compliance [6].

While poor RDT performance with high numbers of false-negative results in the field has been identified as a problem [7–9], false-positive (FP) RDT results are reported less frequently. Currently, South Africa follows the WHO-recommended strategy of using two RDTs to diagnose HIV infection in adults [10]. In the past, the majority of HIV-infected individuals in sub-Saharan Africa had started ART on the basis of two concordantly positive screening and confirmatory RDTs in the context of appropriate clinical or immunological criteria. With universal ART eligibility, there is concern that misdiagnosis of HIV infection and inappropriate ART initiation could be more common because the safety net of the clinical and immunological screenings will be eliminated. While the WHO guidelines recommend retesting prior to ART initiation in order to minimize misclassification of HIV status, the retest policies are often centred on previously RDT-negative individuals. There is little guidance on how large national programmes might go about implementing this additional testing for individuals tested positive by RDTs. There are also few published analyses on the cost implication of such retesting. In South Africa where access to HIV viral load (VL) testing is good, pre-ART VL has been considered as one option for confirmation of HIV infection, but, again, there are few insights into the potential consequences of universal pre-ART VL testing.

In this study, we aim to estimate the proportion of FP RDT through laboratory confirmation using VL and HIV ELISA. We also compare the cost of different retesting strategies with potential inappropriate ART initiation in the test-and-start era.

## Methods

### Study population

This is a retrospective study examining the FP HIV misdiagnosis in a cohort of pregnant women attending antenatal services at a public sector primary care facility in Cape Town, South Africa, between 2013 and 2014. Following local algorithms based on the WHO-recommended two-test strategy, HIV diagnosis in this setting employs two third-generation HIV antibody RDTs: SD Bioline HIV-1/2 (Standard Diagnostics, Kyonggi-do, South Korea) used for screening and the Alere Determine HIV 1/2 (Alere, Waltham, MA, USA) used for confirmation [11].

As part of a larger study of ART in pregnancy [12], we conducted pre-ART VL testing (Abbott RealTime HIV-1) in consecutive HIV-infected pregnant women making their first antenatal clinic (ANC) visit who were not on ART or antiretroviral (ARV) prophylaxis according to self-report. Any pre-ART women who were aviraemic, defined as VL of <50 copies/ml, were further investigated. Some of the women included in this sample were diagnosed with HIV prior to ANC enrolment, while others were diagnosed during the current pregnancy.

### Confirmation of HIV diagnosis

In order to detect the cases of FP HIV misdiagnosis in this population, we consider all women who reported RDT positive and were viraemic during the study as true HIV infection. Women who were identified as aviraemic per their pre-ART VL test and not found to have a subsequent viraemic episode were tested by a fourth-generation HIV ELISA (Enzygnost HIV Intregral4, Siemens, Marburg, Germany) which had a specificity of 99.9% and was optimized in the local laboratory for the purpose of confirmatory testing. Those who were both persistently aviraemic and found to be negative per confirmatory ELISA testing were considered to be HIV uninfected.

### Cost of misdiagnosis

Based on current survival trends in adults in South Africa [13], we estimated that each misdiagnosis would be enrolled in the ARV programme for approximately 30 years. Using the rate of false positivity identified in this analysis, we estimated the costs to identify one erroneous ART initiation using a further RDT, confirmatory HIV ELISA testing, pre-ART VL followed by confirmatory ELISA among aviraemic individuals and confirmatory ELISA for those with CD4 >350 cell/mm^3^. All confirmatory test results are treated as 100% specific. The cost of laboratory testing is derived from the 2015 South African National Health Laboratory Service tariff, and the total programme cost of ART in sub-Saharan Africa is based on a previous published estimate [14]. Uncertainty intervals were calculated based on the 95% confidence interval (CI) of the FP point estimate. We also modelled the cost-comparing confirmatory algorithms above with the cost of treating a misdiagnosis across a spectrum of hypothetical RDT FP rate. Statistical analysis was performed on Stata 12 (Stata Corporation, College Station, USA).

### Ethical approval

The study was reviewed and approved by the University of Cape Town Human Research Ethics Committee (HREC 451/2012) and the Columbia University Medical Center Institutional Review Board (IRB-AAAK8059). All women provided written informed consent prior to participation.

## Results and discussion

This analysis included 952 consecutively enrolled pregnant women who were diagnosed with HIV based on RDT algorithms and who reported no current ART use. The demographic, clinical and laboratory parameters are shown in Table 1. At the time of pre-ART VL testing, the median gestational age for these women was 21 weeks (interquartile range [IQR] 15–27). For women who were diagnosed with HIV prior to the current pregnancy, the median time since HIV diagnosis was 43 months (IQR 21–70). The overall median CD4 cell count was 382 cells/mm^3^ (IQR 255–547), and the median VL among the viraemic women was 4.00 (IQR 3.47–4.58) log copies/ml.

**Table 1.** Study population characteristics.

	Baseline VL ≥50	Baseline VL <50	Total
Number of women	909	43	952
Median age in years (IQR)	27 (24–31)	30 (25–33)	27 (24–32)
Gestational age in weeks (IQR)	21 (15–26)	26 (19–32)	21 (15–27)
Median time since diagnosis (months) (only among women previously diagnosed)	43 (22–70)	34 (15–69)	43 (21–70)
New HIV diagnosis during current pregnancy (%)	479 (53)	25 (58)	504 (53)
Median CD4 cells/mm^3^ (IQR)	373 (250–530)	723 (510–919)	382 (255–547)
Median VL copies/ml (IQR)	10,109 (2956–38,175)	Not Applicable	
Median VL log copies/ml (IQR)	4.00 (3.47–4.58)	Not Applicable	

This table outlines the demographic, pregnancy, immunological and virological characteristics of pregnant women testing HIV positive by RDT. VL: viral load; IQR: interquartile range.

**Figure 1.**
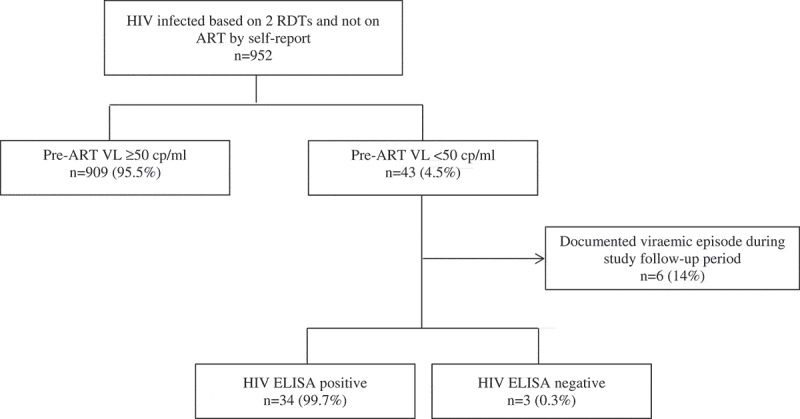
The study flow diagram. Our study examined 952 women entering antenatal care with HIV diagnosis based on two rapid diagnostic tests (RDTs) performed by the routine public sector HIV testing service. Viral load testing, including a pre-ART viral load (VL) test, was performed during their antenatal visits as part of clinical trial participation. Through HIV VL and HIV enzyme-linked immunosorbent assay (ELISA) testing in a subset of these women, we identified the proportion of false-positive RDTs.

**Figure 2.**
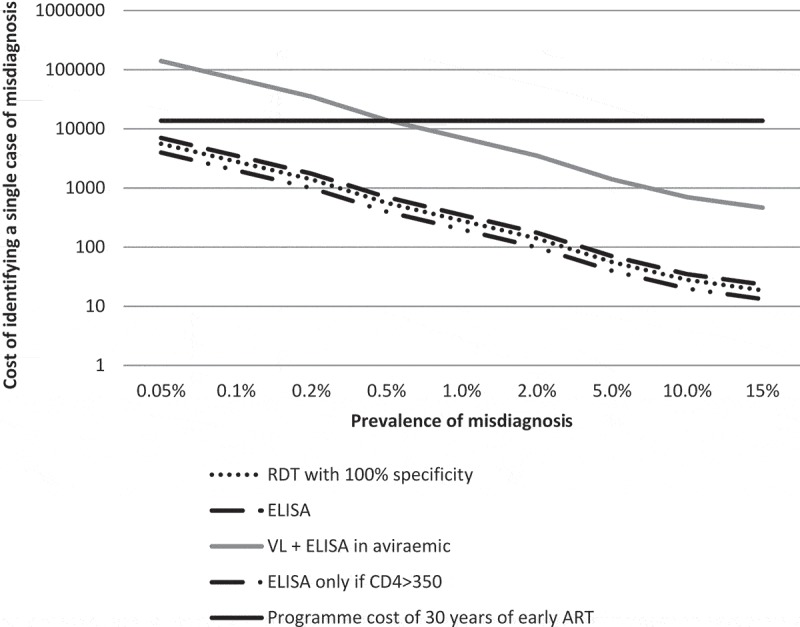
Estimated additional laboratory cost in order to identify a single case of false-positive (FP) rapid diagnostic test (**RDT) by various testing algorithms**. Using a hypothetical RDT FP rate range of 0.05–15%, our model showed that the use of an additional RDT to confirm the initial RDT-based diagnosis will cost $19–$5600 to identify a single case of FP RDT. The cost of using a single ELISA to confirm RDT result ranges from $23 to $7000, while screening with VL followed by ELISA in an aviraemic individual costs between $467 and $140,000. The VL-ELISA algorithm is based on the study data that 4.5% of the pre-ART individuals are aviraemic. Confirming RDT-positive patients with CD4 >350 alone will cost $13–$3976 per positive patient identified. The cost of 30 years of ART and VL monitoring estimate is based on current Clinton Health Access Initiative (CHAI) reference and National Health Laboratory Service (NHLS) prices at $110 per year of tenofovir, lamivudine and efavirenz fixed-dose combination therapy and $20 VL testing per year.

In pre-ART VL testing, 43 women (4.5%) were aviraemic prior to ART initiation or ARV prophylaxis were investigated further as suspected FP from RDTs (Figure 1). Of these, 6 women had detectable virus on subsequent VL measurements; the remaining 37 underwent additional testing using ELISA. Three women were found to be HIV negative by ELISA, representing 7% of all aviraemic women (3/43) and 0.3% (3/952, 95% CI: 0.07–0.9) of all women previously identified as HIV infected by public sector HTS using RDT and who reported not being on ART at the time of entry into antenatal care. Background information on the three cases of misdiagnosis is provided in the supplementary information. The parameters used in the cost comparison and cost modelling are detailed in Table 2. Based on these findings we estimate that immediate use of an ELISA or a third RDT alone as a confirmatory test to identify one patient erroneously initiated on ART would cost $1110 (uncertainty interval, $381-$5382) or $889 ($305-$4306), respectively. By limiting confirmatory testing to individuals with CD4 count >350cells/mm^3^, the cost of ELISA would be further reduced to $631 ($217-3057) while still identifying all false positive results. By contrast, approximately $6397 ($2197-$30,994) would be spent on confirmatory pre-ART VL testing, with further ELISA for aviraemic women, to identify a single patient erroneously initiated on ART. In comparison, based on the current estimate, the total programme cost of 30 years of ART would cost approximately $13710 per misdiagnosis. Modelling the cost comparison across various hypothetical field FP rate suggest that while saving increases with higher FP rates, retest is cost saving even if the FP rate is as low as 0.1% (Figure 2).

**Table 2.** Parameters used for the cost-comparison analysis between the cost of living with the misdiagnosis in the South African ART programme and the cost of HIV status confirmation required to identify a single case of incorrect positive HIV diagnosis

	Value	Source(s)
**Cohort characteristics**		
Proportion of pre-ART individuals with viral load <50 copies/ml	0.045	This study
Proportion of false-positive RDT	0.003	This study
Specificity of various methods of confirmation	100%	Assumption
Proportion of pre-ART individuals with CD4 >350	0.56	This study
Years living with misdiagnosis	30	Johnson et al.
**Cost (USD)**		
Programme cost of early ART per person per year	$457	McGillen et al.
Third RDT	$2.8	South African National Health Laboratory Service tariff
Single ELISA	$3.5	South African National Health Laboratory Service tariff
Single viral load	$20	South African National Health Laboratory Service tariff

ART: antiretroviral treatment; RDT: rapid diagnostic test; ELISA: enzyme-linked immunosorbent assay.

Using a hypothetical RDT FP rate range of 0.05–15%, our model showed that the use of an additional RDT to confirm the initial RDT-based diagnosis will cost $19–$5600 to identify a single case of FP RDT. The cost of using a single ELISA to confirm RDT result ranges from $23 to $7000, while screening with VL followed by ELISA in an aviraemic individual costs between $467 and $140,000. The VL-ELISA algorithm is based on the study data that 4.5% of the pre-ART individuals are aviraemic. Confirming RDT-positive patients with CD4 >350 alone will cost $13–$3976 per positive patient identified. The cost of 30 years of ART and VL monitoring estimate is based on current Clinton Health Access Initiative (CHAI) reference and National Health Laboratory Service (NHLS) prices at $110 per year of tenofovir, lamivudine and efavirenz fixed-dose combination therapy and $20 VL testing per year.

### Discussion

Our data in pregnant women attending antenatal services at a primary care facility in Cape Town, South Africa, highlight the importance of retesting when using RDTs as the sole diagnostic tool, particularly within the new test-and-start paradigm. Among this cohort of pregnant women who were not on ART or receiving ARV prophylaxis, we found that 0.3% of the HIV diagnoses based on two serial RDTs had been incorrect. Compared to other published data on RDT performance in Africa where up to 10% FP rate was reported [15–18], our FP rate appears low in this setting. This could be a signal that there is gradual improvement  in the quality of HTS in the region but also represent the sampling of a relatively well-resourced public sector health system in South Africa. Of note, although these women were enrolled in a clinical trial with rigorous quality assurance (QA) processes, their RDT-based HIV diagnoses were made in routine primary care services prior to trial screening, and thus, we do not believe the trial participation impacted on the FP estimate.

The WHO guidelines on HTS focus on the 5 C’s: consent, confidentiality, counselling, correct results and connection. Within the correct result focus, appropriate use of testing algorithms and retesting before ART initiation are the two major components of ensuring accurate results. The rationale behind the sequential positive RDTs to confirm HIV diagnosis when performed correctly is that the multiplicative effect of combining highly specific RDTs should make misdiagnosis extremely rare. In the WHO laboratory evaluations, the sequential RDT approach achieved >99% positive predictive value when compared to gold standard. However, user errors such as clerical error or poor reading/interpretation can cause non-specificity across various RDTs, despite good assay performance characteristics. Recognizing this potential issue, retesting thus forms a large part of the current WHO strategy to minimize the misdiagnosis. However, many countries’ HIV testing strategies still do not align with the WHO recommendations and many countries, including South Africa, do not have established retesting procedures to confirm the initial screening and confirmatory positive RDTs [10]. In our crude cost comparison, retesting using either an additional RDT or ELISA in order to mitigate FP misclassification is cost saving even if the FP rate is as low as 0.1%. In many LMIC where there are already limited resource of HIV diagnosis, limiting the ELISA confirmation to individuals with CD4 >350 can be a potential strategy which further reduce the cost by 40% while still detecting all FP RDT result. HIV programmes implementing universal ART need to identify a retesting policy that does not delay ART initiation as a matter of urgency as our data suggest that the cost associated with unnecessary lifelong ART and VL monitoring of few individuals misdiagnosed as HIV infected is substantial.

There is no doubt that improving the quality of RDTs should be a key focus of all HTS. Initiatives such as the Rapid Test Quality Improvement Initiative (RTQII) provide quality assurance support and material for proficiency testing. The footprint of these programmes span across many PEPFAR-supported countries and is a key to long-term success of HIV diagnosis in resource-limited settings. It would take a long time and much investment to roll out QA programmes to all the facilities that make use of RDTs to definitively diagnose infection. In South Africa where laboratory infrastructure is good, ELISA performed in a laboratory setting with a quality assurance programme could be invaluable. This form of retesting contributes to not only more accurate diagnoses, but can also be used as a tool to identify primary care facilities that require urgent quality improvement in their RDT programme. It is a far simpler task to roll out quality assurance among laboratories performing ELISA than to have rapid scale up of RTQII coverage of all facilities using RDT to diagnose HIV.

In 2014, the Centers for Disease Control and Prevention (CDC) changed its laboratory diagnostic algorithm to include nucleic acid amplification testing for those samples found to be indeterminate using HIV immunoassays. Similarly, pre-ART VL testing has the potential to be used for confirmatory testing. However, our findings suggest that many women with VL <50 copies were antibody positive. There are many reasons pre-ART individuals may present as aviraemic. A detailed discussion of this phenomenon is beyond the scope of this article, but undisclosed ART use dispensed from a different facility and transient virological control are just two common causes that may confound the use of VL as a confirmatory tool [19–20]. In these aviraemic individuals, further serological testing is required, but our calculation suggests that this tandem VL-ELISA approach is 10 times more costly than simply confirming every positive RDT with ELISA alone.

There are some limitations to our study, the most important of which is the lack of detail around women’s initial RDTs. Reliable documentation of whether any of the positive RDT results were “weakly reactive” could inform potential weaknesses in the current algorithm. This speaks to the fundamental issue around the general lack of formal documentation of RDT HIV diagnoses. In our laboratory confirmation testing, although Western blot was not used to confirm the cases of FP misdiagnosis, the combination of negative ELISA and nucleic acid test is highly specific. For simplicity, we assumed that all confirmation tests, including a further RDT, are 100% specific when calculating the cost of identifying a single FP. In settings where the quality assurance of RDT is poor, our approach would likely underestimate the true cost, and many misdiagnoses can go undetected. More studies with a greater health economic focus are needed to guide the retest policy of many LMIC [21]. Finally, we were not able to assess the greater societal cost of misdiagnosis or the psychosocial impact for the affected individual. Given that they are likely non-trivial, retesting and quality improvement should be a top priority in all HTS, and more resources should be dedicated to ensure that the correct results are provided in our testing facilities.

## Conclusions

In summary, these results suggest that even in the setting where FP HIV RDT diagnoses are relatively uncommon, retesting with additional RDT or ELISA can be cost saving. While testing programmes based on RDT should strive for constant quality improvement, where resources permit, laboratory confirmation algorithms can be cost saving and can play an important role in strengthening the quality of HIV diagnosis in the era of universal ART.

## Notes

To access the supplementary material to this article please see Supplementary Files under Article Tools online.
